# Size Matters: A Single Representation Underlies Our Perceptions of Heaviness in the Size-Weight Illusion

**DOI:** 10.1371/journal.pone.0054709

**Published:** 2013-01-23

**Authors:** Gavin Buckingham, Melvyn A. Goodale

**Affiliations:** The Brain and Mind Institute, Department of Psychology, The University of Western Ontario, London, Ontario, Canada; University of California, Davis, United States of America

## Abstract

In the size-weight illusion (SWI), a small object feels heavier than an equally-weighted larger object. It is thought that this illusion is a consequence of the way that we internally represent objects’ properties – lifters expect one object to outweigh the other, and the subsequent illusion reflects a contrast with their expectations. Similar internal representations are also thought to guide the application of fingertip forces when we grip and lift objects. To determine the nature of the representations underpinning how we lift objects and perceive their weights, we examined weight judgments in addition to the dynamics and magnitudes of the fingertip forces when individuals lifted small and large exemplars of metal and polystyrene cubes, all of which had been adjusted to have exactly the same mass. Prior to starting the experiment, subjects expected the density of the metal cubes to be higher than that of the polystyrene cubes. Their illusions, however, did not reflect their conscious expectations of heaviness; instead subjects experienced a SWI of the same magnitude regardless of the cubes’ material. Nevertheless, they did report that the polystyrene cubes felt heavier than the metal ones (i.e. they experienced a material-weight illusion). Subjects persisted in lifting the large metal cube with more force than the small metal cube, but lifted the large polystyrene cube with roughly the same amount of force that they used to lift the small polystyrene cube. These findings suggest that our perceptual and sensorimotor representations are not only functionally independent from one another, but that the perceptual system represents a more single, simple size-weight relationship which appears to drive the SWI itself.

## Introduction

Despite our impressive repertoire of perceptual abilities, humans tend to make rather imprecise judgments about the veridical weight of an object, instead making relative judgments about how heavy an item is. It is believed that we consciously perceive the weight of an object relative to an expectation (or representation) of how heavy it is likely to be, based on its size, material, and/or other contextual properties. A prime example of the subjective nature of this process comes from illusions of heaviness such as the size-weight illusion (SWI), where a small object feels heavier than a larger, but otherwise similar looking object of the same weight [1]. This powerful illusion does not lessen after prolonged experience with the stimuli, and even persists when the individual is told that the stimuli have the same mass [2], [3].

As alluded to above, it is thought that the SWI is caused by lifters’ incorrect expectations of heaviness [4]. Individuals expect large objects to outweigh similar looking small objects, because they encounter this relationship between size and weight over and over again in the natural world. When lifting objects that induce the SWI, these environmentally-induced expectations are violated (i.e., the larger object does not outweigh the smaller one), leading to the percept that that the small object outweighs the large object. However, the mechanism by which confounded expectations are translated into this perceptual effect remains elusive. One promising line of inquiry suggested that the SWI is caused by lifting errors – specifically from a mismatch between the expected and the actual haptic feedback of the lift [5]. This sensorimotor mismatch explanation has, however, proved unworkable, given that lifters rapidly correct their initial erroneous sensorimotor predictions [2], [6] even though they continue to experience an unchanging perceptual illusion.

The independence of lifting kinetics and heaviness perception has not forced researchers to abandon the notion that the illusion is caused by our expectations. Instead, the concept has been refined to incorporate distinctly adapting representations for the perceptual and sensorimotor systems [7]. In this framework, the sensorimotor system’s predictions are based on a rapidly adapting set of representations, whereas the perceptual system makes use of slowly adapting representations. The rates at which these separate representations adapt are proposed to be a function of necessity. On the one hand, the rapid sensorimotor adaptation facilitates interacting with objects in the world, which may change in mass from lift to lift (i.e., a bottle of water from which you are drinking). On the other hand, the extraordinarily slow adaptation of conscious perception ensures that encountering an unusually-weighted item does not define the new norm for that particular class of object. It is precisely because these perceptual expectations are so resistant to change that the magnitude of the SWI does not diminish with repeated experiences.

There is a growing body of evidence for the roles that cognitive factors play in our conscious perception of heaviness, with numerous reports of weight illusions where top-down factors must play a role. Ellis and Lederman [8] demonstrated that inappropriately-weighted practice golf balls induce a weight illusion in golfers, but not in individuals without golf experience (i.e., who would have no expectations associated with a practice golf ball). Another recent top-down weight illusion comes from Dijker [9], who noted that dolls which would be expected to feel lighter (in this case, a female doll) tended to feel heavier than dolls which were expected to be heavier (a muscular male doll). The most well-studied variants of the SWI are, however, weight illusions caused by manipulating the apparent material properties of the lifted stimuli [10]–[14]. These demonstrations of a so-called ‘material-weight illusion’ (MWI) are, at face value, very similar to the SWI – objects which seem to be made from a light-looking material feel heavier than identically-weighted objects which seem to be made from a heavy-looking material. Furthermore, individuals make lifting errors which reflect their expectations of heaviness, initially lifting the heavy-looking material with a higher force rate than the lighter-looking one. And, like with the SWI, these mistakes which are rapidly corrected with practice [10]. In short, an individual’s cognitive expectations of heaviness can have a wide range of effects on their conscious perception of heaviness.

Although this representation-based view of weight illusions is certainly consistent with much recent work on the topic [15]–[18], it can at this time claim to be only a general description of the SWI [19], with the intricacies of the underlying representations still largely undefined. For example, it is unclear how accurately our perceptual and sensorimotor representations correspond to the *density* of materials (i.e., the slope of the relationship between size and weight of various families of objects). Materials such as polystyrene, in the real world, have a low density and thus require vast increases in volume to become heavy. Metals, on the other hand, have a much higher density and become very heavy with only modest increases in volume. Material properties by themselves have been shown to have clear effects on initial lifting forces and perceptions of heaviness [10]. However, although researchers have suggested that expectations surrounding density are crucial to causing weight illusions [6], this proposition has yet to be tested with stimuli that differ in both actual and apparent density. The predictions are simple: If the SWI is caused by accurate representations of real-world object properties, it follows that lifters should experience a SWI which changes in magnitude as a function of the visual properties (and thus the apparent density) of what we are lifting.

**Figure 1:**
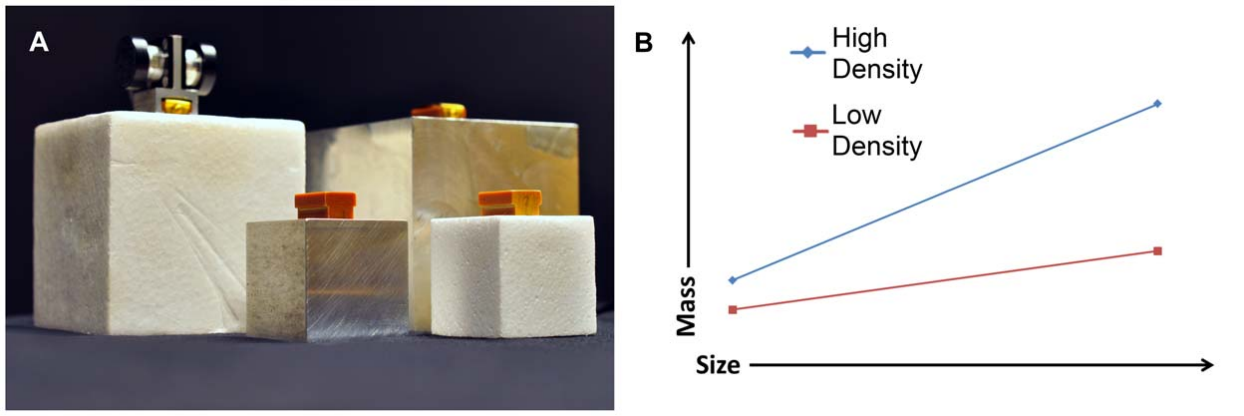
The physical and visual properties of the stimuli. (a) The large and small metal and polystyrene cubes, all adjusted to weigh 700 g and (b) graphical representation of the difference in the size-weight relationship between high density and low density materials.

To this end, we examined individuals’ perception of heaviness and fingertip forces while they picked up identically-weighted, but differently-sized cubes (Figure 1a), one pair appearing to be made from a light-looking material (expanded polystyrene) and the other from a heavier-looking material (aluminum). Based on the prevalent theory regarding the underpinnings of the SWI [2], [7], one would predict that the material properties of the objects will affect the relationship between how small and large exemplars are lifted (i.e., fingertip force errors) and how their weights are perceived (i.e., weight illusions). If, however, the SWI and/or the fingertip forces are unaffected by the markedly different expectations of heaviness that small and large exemplars of different materials should elicit, the notion that cognitive representations are a reflection of the real properties (Figure 1b) of families of objects [7] would have to be refined.

## Materials and Methods

Thirty undergraduate volunteers from the University of Western Ontario took part in this simple lifting experiment. Two participants were removed due to unusual lifting dynamics (grip forces greater than two standard deviations above the mean, leaving a sample of 28 (21 female, 7 male; mean age: 22.5 years, SD: 4.7). Participants were recruited through the university research participation pool in return for course credit or $5 compensation. Subjects gave informed written consent prior to participation. All procedures were conducted according to the principles expressed in the Declaration of Helsinki and were approved by the research ethics board at the University of Western Ontario.

Participants lifted small (5×5×5 cm) and large (10×10×10 cm) cubes made from aluminum (natural density of 2.7 g/cm^3^) and expanded polystyrene (natural density of 0.1 g/cm^3^– see Figure 1b). These cubes were constructed to have identical mass (700 g), which was accomplished by hollowing out the material from which the cubes were made and filling them with various quantities of lead. Functionally, this meant an increase in the overall density of the large polystyrene cube (from 0.1 g/cm^3^ to 0.7 g/cm^3^), the small polystyrene cube (from 0.1 g/cm^3^ to 5.6 g/cm^3^), and the small metal cube (from 2.7 g/cm^3^ to 5.6 g/cm^3^), in addition to a reduction in density of the large metal cube (from 2.7 g/cm^3^ to 0.7 g/cm^3^). There were no visible indications that the natural density of the cubes had been altered in any way (Figure 1a), and care was taken not to handle the cubes in front of the participant before or during the experiment. These adjustments meant that the cubes maintained an approximately central weighting, with a slight bias toward the top of each cube (as none of them had any bottom surface). Each cube had four rubber pads attached to the bottom surface to reduce auditory cues. Prior to lifting any of the cubes, participants were asked to give a numerical rating of how heavy they expected each cube to be, based on its visual appearance alone. This number was then transformed into a percentage of the heaviest value given (in all subjects, this was the value assigned to the large metal cube), in order to account for variations in participants’ range of their numerical values.

A small plastic mount was attached to the top surface of each of the cubes to facilitate the quick attachment and removal of a custom-made handle containing a pair of 6-axis force transducers. These transducers (described in [10]) recorded the grip and load forces in Newton applied to the grasp handles at 1000 Hz. The average of the forces parallel with the surface of the grasp handle were designated as the load force, whereas the average of the forces perpendicular to the surface of the grasp handles were designated as grip force. The force profiles were smoothed with a 14 Hz dual-pass Butterworth filter, and differentiated with a 5-point central difference equation to yield the grip and load forces’ rates of change (GFR and LFR respectively, both measured as Newton per second). Finally, we examined the loading force at the initial peak in its rate of change (LF1^st^), at any time-point after 10% of the overall maximum load force on that trial had been reached (i.e. after it was clear that the lift had been initiated).

In the experiment, participants lifted each of the four cubes 10 times apiece, in one of two different pseudo-random orders (which counterbalanced the material each participant lifted first). These trial orders were organized such that every four lifts participants would have interacted with each of the cubes in a randomized fashion [10]. Participants sat in front of a table wearing PLATO shutter goggles (Translucent Technologies, Toronto, Canada), while the experimenter placed one of the cubes on the table. The shutter goggles were opaque while the cube was placed on the table, so as not to give participants any cues as to its actual mass prior to liftoff. The goggles then opened, at which point participants reached out with their preferred hand and grasped the cube by the handle on its top surface with a thumb and forefinger precision grip and lifted the cube several centimeters off the table surface. Participants were instructed to lift in a ‘smooth, controlled, confident fashion’ to ensure that the lift profile had a natural feed-forward style, as opposed to a probing feedback-style lift. Participants kept the cube held steady for ∼3 seconds, before returning it gently to the table surface. After the lift, participants gave an unconstrained numerical rating of how heavy the cube felt to them [20]. For each participant, this rating was then normalized to a Z-score distribution based on their mean and standard deviation across all trials to account for individual differences in the range of numerical values given by participants during the experiment (N.B. not including the value assigned to the cubes pre-liftoff).

To confirm the presence of the usual perceptual and kinetic errors, the average normalized heaviness ratings and the average GFR, LFR, and LF1^st^ were examined in individual 2 (size) by 2 (material) repeated measures ANOVAs. To examine the specific effect of material cues upon the SWI, we calculated the magnitude of the perceptual SWI for the metal and the polystyrene cubes by subtracting the average large cube rating from the average small cube rating. We then calculated the magnitude of the (opposite direction) size-related force errors by subtracting the average small cube LF1^st^ from the average large cube LF1^st^. We then examined these perceptual and sensorimotor error indices over the course of the 40 lifts and on the initial set of lifts in separate two-tailed paired-sample t-tests, which allowed us to directly examine the effect of size cues in the metal and polystyrene cubes in isolation.

## Results

To verify that participants expected the metal cubes to have a higher density than the polystyrene cubes we calculated an index of expected density based on their initial judgments of how heavy they thought each cube would weigh before they ever picked them up. We calculated this index simply by subtracting the normalized value assigned to the small cube from the normalized value assigned to the large cube for each material. We then compared these expected density indices for the metal and polystyrene sets of cubes with a paired-sample t-test. This analysis confirmed that participants expected the large metal cube to outweigh the small metal cube by a larger amount than they did with the polystyrene set (metal: 32%, polystyrene: 16%; p<.001).

**Figure 2:**
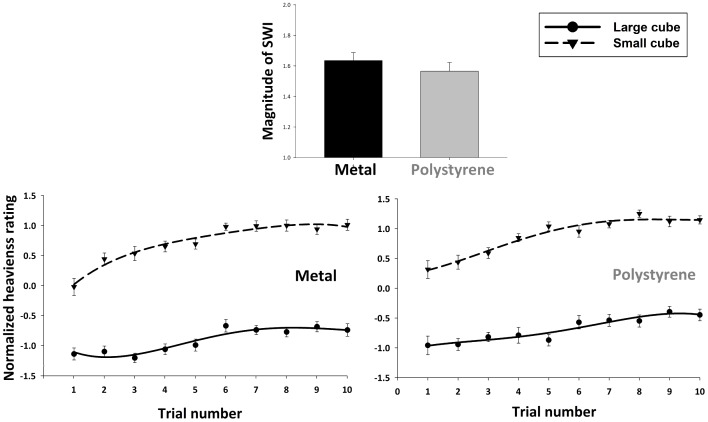
The size-weight illusion as a function of object material. Participants’ reported perceptions of heaviness for the large and small metal and polystyrene cubes. The lower panels show the z-normalized ratings for all the cubes on each trial. These data are fit with 4^th^ order polynomials to indicate the mean trends (no statistics were performed on these curves). The top panels show the average magnitude of the illusion (large cube rating subtracted from small cube rating) over the course of the entire experiment. Error bars indicate between-subject standard error of the mean.

To determine how size and material properties influenced lifters’ perceptions of heaviness we examined the perceptual ratings of heaviness in a repeated-measures ANOVA (Figure 2, bottom). This test revealed significant main effects of size (F(1,27) = 1371.7, p<.001, partial eta^2^ = .98) and material (F(1,27) = 19.6, p<.001, partial eta^2^ = .42). These main effects indicate the presence of a large SWI (average normalized rating of 0.81 for the small cubes and −0.81 for the large cubes) and a smaller MWI (average normalized rating of 0.1 for the polystyrene cubes and −0.1 for the metal cubes) respectively. Crucially, however, these variables did not interact with one another (F(1,27) = 1.22, p = .28), suggesting that SWI and MWI are independent from one another. Paired-sample planned comparisons confirmed that the magnitude of the SWI elicited by the metal cubes did not differ from the polystyrene ones, either on the first trial (p = .51) or over the course of the entire experiment (p = .28; Figure 2, top).

**Figure 3:**
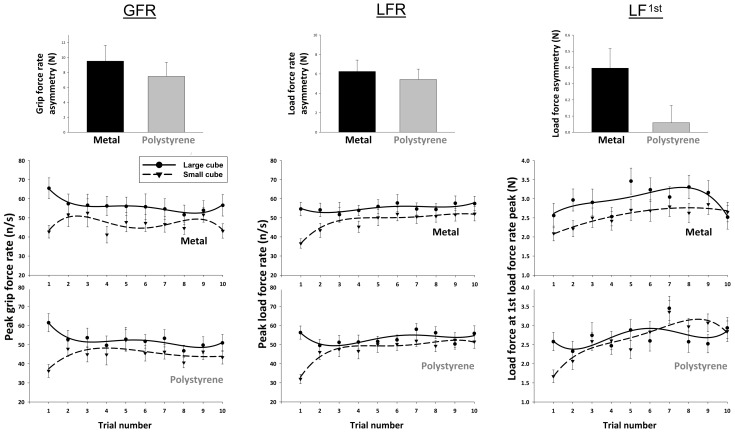
The effects of object size and material on the fingertip forces. The peak grip force rate (GFR – left panels), peak load force rate (LFR – middle panels), and load force at the first peak in load force rate (LF1^st^ – right panels) recorded during lifts of the large and small cubes made from the different materials. The lower panels show the forces for all the cubes on each trial. These data are fit with 4^th^ order polynomials to indicate the mean trends (no statistics were performed on these curves). The top panels show the average magnitude of the size-based errors (force to lift small cube subtracted from force to lift large cube) over the course of the entire experiment. Error bars indicate between-subject standard error of the mean.

To determine how size and material properties influenced lifters’ sensorimotor systems, we analysed peak grip force rate (GFR), peak load force rate (LFR) and load force at the first peak in its rate of change (LF1^st^) with separate repeated-measures ANOVAs (Figure 3, bottom panels). The analysis of GFR revealed a significant main effect of size (F(1,27) = 21.03, p<.001, partial eta^2^ = .43) and material (F(1,27) = 5.19, p<.05, partial eta^2^ = .16. These variables did not, however, interact with one another (F(1,27) = 1.86, p = .18). The analysis of LFR yielded similar results, showing a significant main effect of size (F(1,27) = 39.5, p<.001, partial eta^2^ = .59) and material (F(1,27) = 5.43, p<.05, partial eta^2^ = .17), but no interaction (F(1,27) = 0.43, p = .52). The omnibus statistical analysis of the LF1^st^ revealed a main effect of size (F(1,27) = 8.49, p<.01, partial eta^2^ = .24), but not material (F(1,27) = 1.89, p = .18). The interaction between size and material for LF1^st^ was, however, marginally significant (F(1,27) = 4.15, p = .05, partial eta^2^ = .13), indicating a degree of non-independence in the way that size and material properties influence our lifting forces. Planned comparisons comparing the magnitude of the size-related force errors in LF1^st^ confirmed that this interaction effect was not present during the initial trial (p = .31), but was significant over the course of the entire experiment (p = .05; Figure 3, top right).

## Discussion

In this study we investigated how size and material properties interact with one another when lifting objects and judging their weights. Subjects lifted small and large exemplars of metal and polystyrene cubes which had all been adjusted to weigh 700 g. Although we expected participants to experience a SWI when lifting both the metal and polystyrene cubes, we predicted that these effects might vary as a function of material, given the fundamental relationship between visual material cues and density in the natural world. Remarkably, however, participants experienced SWIs of a very similar magnitude for the metal and polystyrene sets, in stark contrast to the real-world differences in density between metal and polystyrene (see Figure 1b) and the participants’ cognitive understanding of the materials’ properties before the experiment.

The SWI that participants experienced with the metal cubes did not statistically differ from the SWI they experienced with the polystyrene ones indicating that, at the very least, any difference in magnitudes of these illusions was trivially small. Thus, in contrast to their conscious expectations of how heavy the small and large exemplars would be, the perceptual illusion completely was unaffected by the apparent material from which the cubes were made. This finding strongly argues against the intuitive suggestion that the illusion is caused by a contrast with a sophisticated or veridical representation of an object’s likely weight. Put in the current, Bayesian, terminology (see [15]), it is unlikely that the priors which cause the SWI are “based on entire families of objects”, as proposed by Flanagan and colleagues [7]. Instead, our data suggests that the SWI is influenced only to the magnitude of the differences in volume between the stimuli. In other words, rather than being caused by priors based on families of objects, we propose that our conscious perception of how heavy something feels is driven largely by a single expectation of what something a certain size should weigh.

The results of the current study indicate that when judging the weights of objects, our perceptions of heaviness are influenced by a single fixed size-weight relationship, which is used to represent all the possible size-weight families. Although it is difficult to determine where this single prior comes from, one possibility is that it is derived from the average size-weight relationship of *all* the objects that one encounters throughout one’s life. This strategy may be the most optimal compromise between how variable our perceptual experience of an object can be under various conditions (e.g., situational context, fatigue, etc.), and the necessity of making a prediction about the heaviness of an item in the first place. Regardless of how this single representation is formed, it appears to be the mechanism through which the SWI is experienced. It is worth considering why the representation used by sensorimotor system would be comparatively more sophisticated than that used for perceptual judgments. Perhaps the difference stems from the relative importance of each task. Clearly, the task of explicitly judging an object’s weight is a somewhat artificial one, which is unlikely to have played a decisive role in human evolution and/or development. In contrast, and as alluded to above, it is crucial that one considers material properties when attempting to make accurate predictions with fingertip forces, given the potentially disastrous consequences of lifting with too much or too little force. It may be this difference in the relative consequences of consciously perceiving weight incorrectly and lifting objects incorrectly that drives the sensorimotor system to include the extra parameter of likely density (as signaled by visual material cues) in the calculations underlying the initial sensorimotor predictions.

The current work also adds to the growing body of literature suggesting that size information may be the dominant cue in terms of influencing how heavy an object eventually feels when lifted. For one, the MWI is a notably weaker illusion than the SWI, despite the fact that different materials should induce far more disparate expectations of heaviness than the size manipulations in standard SWI tasks (based on the normalized illusion magnitudes for the MWI in [10] with the SWI in [21], we estimate the SWI to be ∼3 times stronger than the MWI). Furthermore, as we have previously noted [10], individuals adapt their fingertip forces to the actual mass of the cubes far more rapidly when lifting cubes that induce the MWI than they do when lifting SWI cubes, despite similar levels of error on the initial trials. Thus, it seems that for the factors surrounding object lifting, an object’s size can be considered as the dominant cue to its weight. This primacy for size cues at the expense of other, high-level, cues may stem from our ability to determine the size of an object directly from the optics of vision, and/or the statistical reliability of the size as a cue to mass *on average* in the natural world.

The other novel aspect of the current work relates to size and material cues’ effects on participants’ predictive fingertip force application. Many current theories of the SWI posit that individuals maintain distinct representations for acting upon objects and perceiving their weights [2], [6], [7], [22]. The current work not only offers strong evidence for this separation of perceptual and sensorimotor representations, but paints a more complex pattern of how sensorimotor prediction is expressed in the fingertip forces than had previously been described. First, we noted that there were clear effects of both size and material on the peak grip and load force rates. The findings with these measures, which are arguably the most commonly reported fingertip force measures, are consistent with our earlier SWI and MWI experiments [10], [21]. These size and material effects for GFR and LFR did not, however, interact with one another. Thus, on the first trial, as well as over repeated lifts, participants made equivalent size-related errors in calibrating their force rates when lifting the metal and polystyrene cubes. This finding appears to suggest that, as with the perceptual illusion, sensorimotor prediction does not accurately reflect the lifted objects’ true properties. In other words, the effects of size and material are independent from one another as far as the sensorimotor system is concerned. By contrast, however, the load force at which the first peak in load force rate occurred (LF1^st^) did show a degree of tuning to the differences in the apparent density of the various objects. This result, combined with the similar-direction trends in the GFR and LFR measures, suggests that there is at least a small degree of tuning to material properties in the sensorimotor system. Perhaps the most surprising aspect of this result is that apparent density is not the sole predictor of sensorimotor prediction when lifting objects. This may indicate that participants only partially include apparent density as a factor in their initial sensorimotor predictions, or that participants were not fooled by our stimuli, never truly expecting the metal cubes to have a higher density than the Styrofoam ones. We consider this latter possibility unlikely, given the pre-liftoff reports for how heavy the subjects expected the cubes to be. While it is difficult to rule out order effects in our paradigm, our findings are consistent recent work by Baugh and colleagues [23], which indicates that apparent density is not the dominant factor in sensorimotor prediction. After a series of lifts with small objects that had different cores from the outward appearance (wood cubes with brass cores and brass cubes with wood cores) they demonstrated that the sensorimotor prediction of the initial lift of a larger cube was not driven entirely by the surface material, but also by the sensorimotor memories encoded from recent lifts.

Another surprising aspect of the current work’s findings was that density appeared to have a small influence sensorimotor over a long series of lifts, rather than the initial lift in isolation. This unexpected result means that, when lifting objects that appear to be made from a heavy material, individuals struggle to adequately adapt their loading forces to the actual weights of the objects. Although this conclusion does have to be tempered by the borderline statistical significance and small size of the effect, it does parallel observations from a recent study [21] showing that load force tends not to adapt with the same degree of precision as grip and load forces’ rates of change. It appears that this failure to adapt may be mediated by the material from which an object appears to be made, with the polystyrene cube showing comparatively rapid load force adaptation.

To sum up, the results of the current study have confirmed that distinct representations underpin the predictions made when lifting SWI-inducing objects and judging their weights. Furthermore, the representation underpinning our perception of heaviness appears to operate on a much simpler principle than previously thought: a fixed increase in size will yield a fixed increase in weight, regardless of apparent density. We suggest that the SWI is caused by a single representation derived from the average of our overall experience with all liftable objects encountered throughout our lives.
